# Clinical characteristics of *Pneumocystis *pneumonia in non-HIV patients and prognostic factors including microbiological genotypes

**DOI:** 10.1186/1471-2334-11-76

**Published:** 2011-03-25

**Authors:** Yasufumi Matsumura, Yuichiro Shindo, Yoshitsugu Iinuma, Masaki Yamamoto, Michinori Shirano, Aki Matsushima, Miki Nagao, Yutaka Ito, Shunji Takakura, Yoshinori Hasegawa, Satoshi Ichiyama

**Affiliations:** 1 Department of Clinical Laboratory Medicine, Kyoto University Graduate School of Medicine, Kyoto, Japan; 2 Department of Respiratory Medicine, Nagoya University Graduate School of Medicine, Nagoya, Japan; 3 Department of Infectious Diseases, Kanazawa Medical University, Kanazawa, Japan; 4 Department of Infectious Diseases, Osaka City General Hospital, Osaka, Japan; 5 Department of Respiratory Medicine, Kyoto University Graduate School of Medicine, Kyoto, Japan

## Abstract

**Background:**

The number of patients with non-HIV *Pneumocystis *pneumonia (PCP) is increasing with widespread immunosuppressive treatment. We investigated the clinical characteristics of non-HIV PCP and its association with microbiological genotypes.

**Methods:**

Between January 2005 and March 2010, all patients in 2 university hospitals who had been diagnosed with PCP by PCR were enrolled in this study. Retrospective chart review of patients, microbiological genotypes, and association with 30-day mortality were examined.

**Results:**

Of the 82 adult patients investigated, 50 patients (61%) had inflammatory diseases, 17 (21%) had solid malignancies, 12 (15%) had hematological malignancies, and 6 (7%) had received transplantations. All patients received immunosuppressive agents or antitumor chemotherapeutic drugs. Plasma (1→3) β-D-glucan levels were elevated in 80% of patients, and were significantly reduced after treatment in both survivors and non-survivors. However, β-D-glucan increased in 18% of survivors and was normal in only 33% after treatment. Concomitant invasive pulmonary aspergillosis was detected in 5 patients. Fifty-six respiratory samples were stored for genotyping. A dihydropteroate synthase mutation associated with trimethoprim-sulfamethoxazole resistance was found in only 1 of the 53 patients. The most prevalent genotype of mitochondrial large-subunit rRNA was genotype 1, followed by genotype 4. The most prevalent genotype of internal transcribed spacers of the nuclear rRNA operon was Eb, followed by Eg and Bi. Thirty-day mortality was 24%, in which logistic regression analysis revealed association with serum albumin and mechanical ventilation, but no association with genotypes.

**Conclusions:**

In non-HIV PCP, poorer general and respiratory conditions at diagnosis were independent predictors of mortality. β-D-glucan may not be useful for monitoring the response to treatment, and genotypes were not associated with mortality.

## Background

*Pneumocystis jirovecii *pneumonia (PCP) is widely known as an opportunistic infection in human immunodeficiency virus (HIV)-infected patients. The introduction of chemoprophylaxis and highly active antiretroviral therapy has reduced the incidence of HIV PCP in recent years [1,2]. In contrast, PCP in non-HIV immunocompromised patients is increasing as the number of patients receiving transplantation, immunosuppressive therapy, and antitumor chemotherapeutic agents continues to grow [1].

For years, the standard method for laboratory diagnosis of PCP was the visualization of *P. jirovecii *by microscopy in bronchoalveolar lavage (BAL) fluid or induced sputum. It is known that non-HIV patients often develop PCP with a lower parasite burden than HIV patients [3], which may result in a false negative microscopic determination [4]. Although immunofluorescent antibody stain has improved the sensitivity compared to Gomori methenamine silver or Giemsa stains [5], a more sensitive polymerase chain reaction (PCR) test has recently been employed [6,7]. However, being PCR-positive does not necessarily indicate a diagnosis of PCP. *Pneumocystis* colonization, defined as detection of the organism or its DNA without signs or symptoms of pneumonia, has been reported irrespective of immunosuppressive conditions [8]. Furthermore, PCR-positive patients with pulmonary infiltrates diagnosed as a pneumonia other than PCP were also considered to have colonization of *P. jirovecii *[4,9]. Without microscopy-positive patients, differentiation between PCP and colonization can only be done by clinical diagnosis. For adjunctive diagnosis, (1→3) β-D-glucan tests have also been used [10-12]. Because most of the studies on non-HIV PCP have been based on microscopic diagnosis, its clinical characteristics, including the β-D glucan test in PCR-diagnosed non-HIV PCP patients, are still not well described.

Strain analysis of *P. jirovecii *had been conducted by molecular genotyping based on nucleotide sequence variations because of the inability to culture. Studies mainly on HIV PCP have targeted the mitochondrial large-subunit rRNA (mt LSU rRNA) and internal transcribed spacer (ITS) regions of the nuclear rRNA operon, and the dihydropteroate synthase (DHPS) gene [13]. Mt LSU rRNA and ITS have been used to analyze the cluster of PCP, the route of transmission, and association of severity [13,14]. The DHPS mutation is thought to be associated with failure of treatment and prophylaxis [15,16]. However, molecular epidemiology and the clinical relationship with non-HIV PCP have been described in only small-scale studies.

In this study, we describe the clinical characteristics of non-HIV PCP based on PCR diagnosis. This includes the underlying conditions, laboratory findings such as β-D glucan tests, complications, and the association of prognostic factors. We also evaluate the clinical significance of microbiological genotypes.

## Methods

### Study site and population

We retrospectively reviewed all consecutive non-HIV patients tested by *pneumocystis* PCR analysis between January 2005 and March 2010 at 2 tertiary care university hospitals, Kyoto University Hospital and Nagoya University Hospital, in Japan. After reviewing the medical records for all patients tested, diagnoses of PCP were made if the patients met all of the following criteria: new ground glass opacities in chest computed tomography, positive PCR targeting of mt LSU rRNA, and clinical suspicion of PCP by an attending physician defined as presumptive treatment for PCP. Presumptive treatment occurred when PCR results were pending and continued until resolution or death. Only patients with the first episode of PCP were included. If multiple samples were taken for a patient, the samples were taken in the following order: BAL fluid, sputum, and oral wash. The Ethics Committee of Kyoto University Graduate School and Faculty of Medicine (E-356) and the Institutional Review Board of Nagoya University Graduate School of Medicine (641) approved this study and waived the need for obtaining informed consent from each patient.

### Data collection

Clinical information acquired by medical chart review included underlying diseases, immunosuppressive therapies during the previous month, PCP prophylaxis, clinical symptoms, laboratory values, Sequential Organ Failure Assessment (SOFA) score [17], anti-PCP treatment, complications, invasive fungal infections, and 30-day mortality. The daily dosage of corticosteroids was expressed as the prednisolone equivalent (1 mg of prednisolone equals 0.8 mg of methylprednisolone equals 1 mg of prednisone). Hypoxemia was defined as arterial PaO_2 _< 70 mm Hg in room air or a requirement for supplemental oxygen. Invasive fungal infection was diagnosed according to the European Organization for Research and Treatment of Cancer criteria [18].

### β-D-glucan assay

Plasma β-D-glucan was measured with the β-glucan test WAKO (Wako Pure Chemical Industries; Tokyo, Japan). Plasma samples were collected before and after PCP treatment. The assay was performed as a clinical routine at our institution on the same day when the plasma was obtained.

### PCR detection and genotyping

DNA was extracted using the QIAamp DNA mini kit (Qiagen; Hilden, Germany). Molecular detection of *P. jirovecii *was carried out by single round PCR amplification of mt LSU rRNA [19].

DHPS genotypes were determined by nested PCR and RFLP analysis [20], based on codon 55/57 mutations: wild type (Thr/Pro), single mutant (Ala/Pro or Thr/Ser), and double mutant (Ala/Ser). Mt LSU rRNA genotypes were determined by direct sequencing at nucleotides 85 and 248: genotype 1 (C/C), 2 (A/C), 3 (T/C), 4 (C/T), and 5 (C/T). When mixed genotypes were suspected, amplification products were cloned and then 5 clones were randomly selected and sequenced. ITS regions were amplified by nested PCR [21], and the 5 clones were analyzed using scores described elsewhere [22-25]. If multiple haplotypes (combinations of ITS1 and ITS2 types) were detected from 1 sample, mixed-type were considered only when both haplotypes were detected in at least 1 single-type sample.

To investigate the relationship with severity or outcome, we compared each genotype with hypoxemia, SOFA score, mechanical ventilation, and 30-day mortality.

### Statistical analysis

Categorical variables were compared using Fisher's exact test. Continuous variables were compared using the Mann-Whitney U test. β-D-glucan values under the commercial upper limit of the normal value of 11 pg/mL were considered to be 1.1 pg/mL when comparing the values before and after treatment. To determine the association of independent variables with 30-day mortality, a stepwise logistic regression analysis was performed. Variables with a P-value of less than 0.10 on univariate analyses were included in the multiple regression model with step forward analysis. P < 0.05 was considered statistically significant. We performed our statistical analyses using R version 2.9.2 (R foundation for Statistical Computing; http://www.r-project.org).

## Results

**Figure 1:**
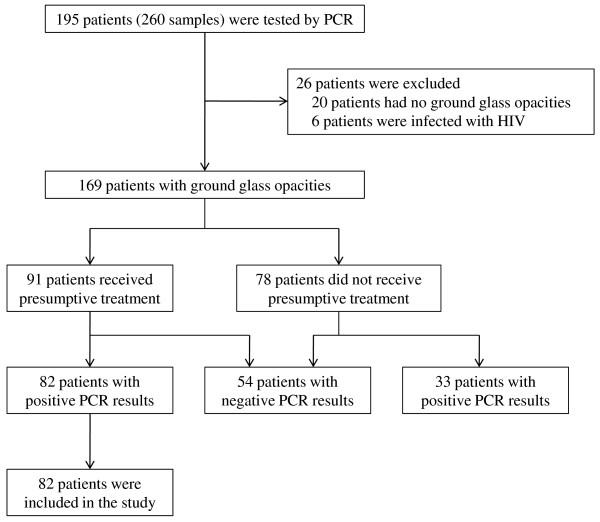
**Inclusion of patients**. PCR: polymerase chain reaction; HIV: Human Immunodeficiency Virus.

As detailed in Figure 1, 260 samples from 195 patients were tested by PCR during the 5-year observation period, and 82 patients fulfilled the inclusion criteria. Among 78 patients who did not receive presumptive treatment, 2 patients received treatment after PCR results turned out to be positive. The final diagnoses in 33 patients with no presumptive treatment and positive PCR results were infection other than PCP (bacterial pneumonia, n = 5; aspergillosis, n = 3; atypical pneumonia, n = 1; tuberculosis, n = 1; viral pneumonia, n = 1), interstitial pneumonia associated with collagen diseases (n = 8), drug-induced pneumonia (n = 5), miscellaneous (n = 4), and unknown etiology (n = 5).

Of 82 PCP patients, 64 patients (39 males) were from Kyoto University Hospital and 18 (12 males) were from Nagoya University Hospital. The median age was 64 years (range, 19-82 years). Respiratory samples that were obtained for PCR were sputum in 41 patients, BAL fluid in 35, and oral wash in 6. Sputum or oral wash were simultaneously obtained in 9 patients who underwent bronchoscopy, which were all PCR-positive. Some of the samples underwent microscopic analysis using the Gomori methenamine silver stain. Eight of 33 (24%) samples of BAL fluid and 2 of 31 (6%) of sputum were microscopically positive.

### Clinical Characteristics

Underlying diseases and conditions of patients are listed in Table 1. All patients had received some kind of immunosuppressive therapy; 74 patients (90%) received corticosteroids or immunosuppressive agents and the other 8 patients received antitumor chemotherapy alone. Table 2 shows that corticosteroids were administered in 65 patients (79%), with a median dose of prednisone of 13 mg/day. Twenty-four patients (29%) had received corticosteroids less than 10 mg/day and 7 (9%) patients for a period of less than 1 month. Forty-one (50%) patients had received immunosuppressive agents other than corticosteroids, with methotrexate as the most commonly used agent (20 patients, 24%), followed by cyclosporin (10 patients, 12%), tacrolimus (8 patients, 10%), and the antitumor necrosis factor-α drugs, infliximab and etanercept (6 patients, 7%). Twenty-two patients (27%) had received antitumor chemotherapy. Prophylactic therapy was used in 3 patients (4%). Two patients had received TMP-SMX, at a dose of 1 single-strength tablet once daily for 5 months and 3 inconsecutive days weekly for 2 weeks, respectively. Another patient had received aerosolized pentamidine at a dose of 300 mg once monthly for 2 months. No patient had a neutrophil count under 500/mm^3^. Lactate dehydrogenase (LDH) was above the upper limit of the normal value (>241 IU/L at Kyoto University and >229 IU/L at Nagoya University) in 76 patients (93%). β-D-glucan was elevated in 65 (80%) of 81 patients. Probable invasive pulmonary aspergillosis was concomitant with 5 PCP patients who had inflammatory diseases. In addition to ground glass opacities, 2 patients had nodules without a halo sign, 2 had consolidation, and 1 had an air-crescent sign. All 5 patients were positive by enzyme-linked immunosorbent assay for the detection of *Aspergillus *galactomannan at a threshold of 1.0. One patient was also positive with culture of *Aspergillus fumigatus*. Three patients developed aspergillosis after PCP diagnosis, 1 simultaneously, and 1 before. Four patients had elevated levels of β-D-glucan before and after PCP treatment. As aspergillosis can influence β-D-glucan levels, we excluded those patients with aspergillosis from the analysis of β-D-glucan monitoring. No other invasive fungal disease or mycobacterial infection was identified.

**Table 1:** Patient demographics at diagnosis of pneumocystis pneumonia in 82 patients

Characteristics	**No**.	(%)
Age, years (range)	64	(19-82)
Male sex	51	(62%)
Underlying disease		
Inflammatory disease	50	(61%)
Rheumatoid arthritis	17	(21%)
Systemic lupus erythematosus	10	(12%)
Vasculitis	7	(9%)
Inflammatory myopathy	4	(5%)
Pemphigus or pemphigoid	3	(4%)
Miscellaneous	11	(13%)
Solid malignancy	17	(21%)
Lung cancer	9	(11%)
Other tumors	8	(10%)
Hematological malignancy	12	(15%)
Malignant lymphoma	6	(7%)
Myelodysplastic syndrome	3	(4%)
Miscellaneous	3	(4%)
Organ transplantation	6	(7%)
Kidney	3	(4%)
Liver	2	(2%)
Bone marrow	1	(1%)
Fulminant hepatitis	2	(2%)
Pulmonary disease other than lung cancer	18	(22%)
Interstitial pneumonia	9	(11%)
Chronic obstructive pulmonary disease	4	(5%)
Miscellaneous	5	(6%)
Current or ex-smoker	28	(34%)

Some patients had 2 or more underlying diseases or conditions.

**Table 2:** Clinical characteristics

	All patients		Survivors		Non-survivors		
Characteristics	(n = 82)		(n = 62)		(n = 20)		P value
Corticosteroids	65	(79%)	48	(77%)	17	(85%)	0.55
Daily dose, mg	13	(10-23)	13	(10-25)	15	(11-20)	0.39
Less than 10 mg/day	24	(29%)	20	(32%)	4	(20%)	0.40
Total duration, days	152	(66-943)	153	(67-889)	147	(38-2346)	0.95
Less than 1 month	7	(9%)	4	(6%)	3	(15%)	0.35
Immunosuppressive agents	41	(50%)	35	(56%)	6	(30%)	0.07
Antitumor chemotherapy	22	(27%)	14	(23%)	8	(40%)	0.15
PCP Prophylaxis	3	(4%)	1	(2%)	2	(10%)	0.15
Lower respiratory symptoms	74	(90%)	55	(89%)	19	(95%)	0.67
Fever	56	(68%)	42	(68%)	14	(70%)	1.00
Hypoxemia	57	(70%)	39	(63%)	18	(90%)	0.03
PaO_2_/FiO_2 _ratio (n = 50)	277	(168-341)	304	(186-356)	183	(108-318)	0.07
SOFA score	2	(1-4)	2	(1-3)	3	(1-5)	0.11
Laboratory findings							
Neutrophils,/mm^3^	6750	(5000-9525)	6250	(4975-8700)	8100	(5475-15425)	0.06
Lymphocytes,/mm^3^	545	(400-1100)	750	(475-1225)	500	(200-675)	0.03
Thrombocytes,/mm^3^	15.9	(11.5-24.7)	16.2	(12.2-25.3)	14.3	(7.3-24.7)	0.20
C-reactive protein, mg/dL	6.4	(2.5-10.7)	5.6	(2.5-9.6)	8.5	(2.1-13.6)	0.18
Albumin, mg/dL	2.8	(2.4-3.2)	2.9	(2.5-3.3)	2.3	(2.2-2.9)	0.007
Creatinine, mg/dL	0.9	(0.6-1.3)	0.9	(0.6-1.2)	0.9	(0.6-1.6)	0.53
Blood urea nitrogen, mg/dL	21	(16-31)	20	(15-27)	30	(16-63)	0.25
Total bilirubin, mg/dL	0.6	(0.4-0.9)	0.6	(0.4-0.8)	0.7	(0.5-0.9)	0.43
Lactate dehydrogenase, IU/L	394	(303-501)	368	(291-485)	456	(366-662)	0.007
LDH > upper limit of normal value	76	(93%)	56	(90%)	20	(100%)	0.33
β-D-glucan (n = 81), pg/mL	39.7	(14.5-207.5)	36.3	(15.0-204.7)	53.6	(11.3-353.7)	0.50
β-D-glucan (n = 81) >11 pg/mL	65	(80%)	49	(79%)	15	(79%)	1.00
							
Initial Treatment							
TMP-SMX	77	(94%)	58	(94%)	19	(95%)	1.00
Drug change	19	(25%)	13	(22%)	6	(32%)	0.54
Pentamidine	5	(6%)	4	(6%)	1	(5%)	1.00
Drug change	1	(20%)	1	(25%)	0	(0%)	1.00
Adjunctive steroid therapy	60	(73%)	44	(71%)	16	(80%)	0.57
Anti-cytomegalovirus therapy	23	(28%)	15	(24%)	8	(40%)	0.25
Mechanical ventilation	22	(27%)	9	(15%)	13	(65%)	<0.001
Pneumothorax	4	(5%)	1	(2%)	3	(15%)	0.04
Invasive pulmonary aspergillosis	5	(6%)	1	(2%)	4	(20%)	0.01

Data are presented as the No. (%) or median (interquartile range). Steroid dosages are expressed as corresponding amounts of prednisolone. Categorical variables were compared using Fisher's exact test, and continuous variables were compared using the Mann-Whitney U test.

PCP: pneumocystis pneumonia; SOFA: Sequential Organ Failure Assessment; LDH: lactate dehydrogenase; TMP-SMX: trimethoprim-sulfamethoxazole.

**Figure 2:**
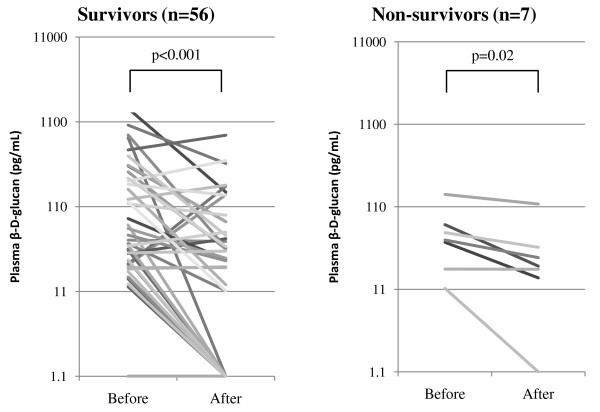
**Plasma β-D-glucan levels before and after treatment**. Values after treatment were measured at day 21 in survivors and at day 17 in non-survivors (median value). Values after treatment were significantly reduced in both groups by the Mann-Whitney U test. Values under the commercial cutoff value of 11 pg/mL were considered to be 1.1 pg/mL.

Levels of β-D-glucan after treatment were significantly reduced in both groups of 56 survivors and 7 non-survivors (Figure 2). There were 10 survivors (18%) and 1 non-survivor (14%) whose β-D-glucan increased after treatment. Normalization occurred in only 33% of survivors (15 of 46) and in 14% of non-survivors (1 of 7).

The crude 30-day mortality was 24% (20 deaths). Table 2 shows the factors that were significantly associated with mortality in univariate analysis, such as hypoxemia (p = 0.03), low lymphocytes (p = 0.03), low serum albumin (p = 0.007), high LDH (p = 0.007), mechanical ventilation (odds ratio [OR], 10.9; 95% confidence interval [CI], 3.4 to 34.9), pneumothorax (OR, 10.8; CI, 1.1 to 110.2), and invasive pulmonary aspergillosis (OR 15.3; CI, 1.6 to 146.1). Multivariate analysis revealed that low serum albumin and mechanical ventilation were independent predictors of mortality (Table 3).

**Table 3:** Clinical features associated with 30-day mortality in multivariate logistic regression analysis

Variable	Odds ratio	**95% C.I**.	P value
Immunosuppressive agents other than corticosteroids	0.31	0.07-1.41	0.13
Lymphocyte, per 100/mm^3^	1.06	0.97-1.16	0.19
Albumin, per 0.1 mg/dL	0.85	0.73-0.99	0.03
Lactate dehydrogenase, per 100 IU/L	1.65	1.00-2.73	0.05
Mechanical ventilation	5.16	1.25-21.32	0.02
Invasive pulmonary aspergillosis	20.07	0.94-426.88	0.05

### Genotyping

Respiratory samples from 56 (68%) patients were subjected to genotyping (Table 4). The other 26 samples taken before January 2008 at Kyoto University and September 2008 at Nagoya University were not stored. The mt LSU rRNA genotype analyses were successful in all of the 56 patients. Genotype 1 was most common (30%), followed by genotype 4 (27%), genotype 2 (18%), genotype 3 (13%), genotype 5 (2%), and mixed genotype (11%). The DHPS gene was amplified in 53 patients (95%). Of the patients without prophylaxis, only 1 patient had the double mutation and all the others were wild-type. The ITS gene was amplified in 31 patients (55%). We identified 9 unique ITS haplotypes from a single-type sample. Genotypes Ci, Ea, Ef, Ei, Hg, and Jf were detected only in mixed-type samples, each from 1 patient. The most prevalent ITS types were Eb (9 patients, 29%), followed by Eg (6 patients, 19%), and then Bi (5 patients, 16%). Mixed-types were detected in 5 patients (16%). The ITS genotype and epidemiological distribution showed no cluster outbreak in this study period. None of the genotypes of 3 genetic loci were associated with hypoxemia, SOFA score, mechanical ventilation, or mortality in univariate analysis. The DHPS gene was excluded from analysis because only 1 patient had the mutant genotype.

**Table 4:** Genotypes of *Pneumocystis jirovecii *in 56 patients

Genetic locus	Total numbers tested	Genotype					
mt LSU rRNA	56	1	2	3	4	5	Mixed
		17 (30)	10 (18)	7 (13)	15 (27)	1 (2)	6 (11)

ITS	31	Eb	Eg	Bi	Ai, Ce, "H"r, Ir, Iu_3_, U_1_e	Mixed	
		9 (29)	6 (19)	5 (16)	1 (3)	5 (16)	

Data are presented as No. (%). mt LSU r rRNA, mitochondrial large subunit; based on nucleotide 85/248, genotypes 1, C/C; 2, A/C; 3, T/C; 4, C/T; 5, A/T. ITS, internal transcribed spacer. If multiple haplotypes (combinations of ITS1 and ITS2 types) were detected from 1 sample, mixed-type were considered only when both haplotypes were detected in at least 1 single-type sample.

## Discussion

PCP is diagnosed by identifying *P. jiroveci *in respiratory specimens, but it is often difficult to obtain BAL samples in non-HIV patients with severe and rapidly progressive disease [3,26]. Highly sensitive PCR allowed us to detect *P. jirovecii *in samples with negative direct examination tests and in non-invasive samples, such as induced sputum [4] or oral wash [27]. Although PCR for *P. jiroveci *can be positive in asymptomatic colonized patients, PCP therapy is recommended in patients with clinical suspicion of PCP and positive PCR results [7]. According to these backgrounds, our diagnostic criteria included presumptive treatment due to strong suspicion by a clinician for PCP, in addition to the PCR and imaging studies. We excluded 2 patients for whom treatment was initiated after the PCR result. Because it usually takes 3 to 10 days to get PCR results in our practice, a delay in treatment would have some effect over outcomes. PCR-positive patients without treatment had clinical diagnoses other than PCP. PCR-negative patients irrespective of receipt of treatment were also excluded, because PCR in non-HIV patients has a quite high negative predictive value [4].

The main risk factors for PCP are deficiencies in cellular immunity and the use of immunosuppressive agents, especially corticosteroids. Similar to previous reports [28,29], even with a low dose or short duration of corticosteroids, our patients developed PCP. In addition to corticosteroids, the introduction of high-intensity immunosuppressive regimens such as anti-tumor necrosis factor-α drugs has increased in inflammatory diseases. Post-marketing surveillance of anti-tumor necrosis factor-α drugs revealed a much higher incidence of PCP in Japan than in the US [30]. Six PCP patients (7.3%) in this study received anti-tumor necrosis factor-α drugs. Reported attack rates of PCP in non-HIV patients not receiving prophylaxis are highest in hematological malignancies and transplantation [28,31]. In our study, almost two-thirds of PCP patients had inflammatory diseases and fewer patients had hematological malignancies or transplantation. This may be because PCP prophylaxis is common in hematological malignancies or transplantation, but not in inflammatory diseases, and the number of patients with inflammatory diseases in the general population is larger.

Elevated serum LDH levels have been noted in patients with PCP [11] but were not specific. β-D-glucan was reported to be elevated in 96% of 111 HIV patients with microscopic diagnosis of PCP [10] and 78% of 18 non-HIV patients with a PCR-based diagnosis [32]. The latter value is almost the same as our result (80%). Some reports have found high β-D-glucan to be a predictor of death [32,33], while others have not [34,35]. The β-D-glucan value did not differ between survivors and non-survivors in our study (Table 2). In a study of 21 non-HIV PCP patients of whom 2 died within 30 days, it was reported that β-D-glucan a median of 3 days after treatment had significantly decreased [35]. In our study, β-D-glucan in survivors and non-survivors was separately analyzed, and in both groups it was significantly reduced after treatment. However, normalization occurred only in 33% of survivors, and in some patients from both groups, β-D-glucan increased after treatment. In a report of 42 HIV PCP patients, normalization after 3 weeks of successful treatment occurred in only 17% of patients, and elevated levels were found in 21% of patients [10]. These results suggest that a decrease of β-D-glucan simply corresponds with treatment, but does not predict treatment response. Since β-D-glucan is a cell wall component of *P. jirovecii*, the level of β-D-glucan may reflect the organism burden directly, not the severity of PCP. Limper et al. demonstrated that mortality was not associated with organism burden, but rather the number of neutrophils in the lower respiratory tract [3]. When β-D-glucan increases compared to the level before treatment, it may not indicate deterioration of PCP.

Non-HIV immunocompromised patients often develop invasive aspergillosis [36]. We found 5 patients who developed probable aspergillosis. Although *Aspergillus *has not been generally recognized as a co-pathogen of *P. jirovecii*, it is possible that it is because both PCP and aspergillosis develop with the use of immunosuppressive agents [28,29,36]. Aspergillosis was found to be a significant predictor of death in univariate analysis and almost reached significance in multivariate analysis. Aspergillosis should be considered when there is a poor treatment response to PCP.

Twenty-four percent of our patients died, which is similar to the reported mortality rate of 19 to 40% [1,29,37]. We found that albumin and mechanical ventilation were independent predictors. These factors have been reported previously [29,37].

The epidemiology of PCP in large numbers of non-HIV patients separate to that of HIV patients has never been reported. From a previous small Japanese study, 2 of 8 non-HIV patients had the DHPS mutation [38]. Our patients had a very low rate of DHPS mutations, which cannot be generalized for non-HIV patients, because mutant rates in HIV patients vary widely (3% to 69%) [16]. In addition, the prophylactic use of TMP-SMX may be associated with the emergence of DHPS mutant strains in HIV patients [39]. One possible explanation of the low rate of DHPS mutants in our patients is the rarity of prophylaxis.

There is little information about the clinical background of the mt LSU rRNA genotypes. The only study describing the clinical relationships in 53 HIV and 7 non-HIV patients found no association [40]. We also found no association in our patients. The largest study that has investigated the genotypes of non-HIV PCP included 17 patients from 3 PCP clusters in Sweden [41]. Genotypes 1, 2, 3, and mixed-types were found in 5, 2, 3, and 7 patients, respectively. Other studies for HIV PCP, non-HIV PCP, and non-HIV patients without pneumonia found that genotypes 1 or 2 were the most prevalent, and genotype 4 was the least prevalent [20,40,42-45]. Genotype 1 was the most prevalent in our study, which is consistent with those reports. However, it is inconsistent with regard to genotype 4, which was the second prevalent type in this study.

The clinical correlation with the ITS genotypes has been conflicting [14,46]. We did not find a correlation. Some studies have focused on the epidemiology of the ITS genotype in non-HIV PCP [40,47], but the number of patients was less than 10. We investigated 31 patients and found Eb, Eg, and Bi from multiple patients as a single haplotype. Mixed infections were detected in 16%. Genotypes Eb, Eg, Bi, and Ne are reported to be common in HIV PCP, and mixed infections are reported in 5-79% [20,22-24,40,48]. Those previous studies overestimated the mixed infections, because many of the less common haplotypes resulted from *in vitro *recombinations of globally common ITS haplotypes present in the same sample [21].

Our study is retrospective, and it has several limitations. One limitation is the heterogeneity of the respiratory sample and underlying clinical conditions. More than half of the samples obtained for PCR were not BAL fluid, and not all of the samples underwent microscopic analysis. Of the PCR-diagnosed PCP, microscopic examinations were positive only in 24% of BAL fluid and in 6% of sputum. One possible reason for the low positive rate is that Gomori methenamine silver stain has less sensitivity than an immunofluorescent antibody stain [5]. The underlying clinical conditions and stage of disease might have varied, which could be uncontrolled confounders of the cause of death. Another limitation is that our study may still lack the statistical power to determine a clinical relationship between genotypes and outcome, although it is the largest to date on genotypes of non-HIV PCP.

## Conclusions

This is the largest study to date that describes the clinical characteristics of non-HIV PCP based on PCR diagnosis. We found that inflammatory disease was the most common underlying condition, and all of the patients received immunosuppressive agents or chemotherapeutic agents. β-D-glucan may not be useful for monitoring the response to treatment. Predictors of death were similar to previous reports, except that invasive pulmonary aspergillosis was a predictor of death in univariate analysis. During treatment, invasive pulmonary aspergillosis should be noted carefully. Microbiological genotypes of non-HIV PCP were also described in the largest population, in which there were few DHPS mutation and no relationship with the clinical characteristics. The prevalence of ITS and mt LSU rRNA genotypes in non-HIV patients did not seem to be significantly different when compared to reports for HIV patients. Further investigation is needed to evaluate microbiological genotypes from a clinical point of view.

## Competing interests

The authors declare that they have no competing interests.

## Authors' contributions

YM conceived of the study, participated in its design, reviewed the medical records, carried out the genetic studies, performed the statistical analysis, and drafted the manuscript. YS and YH participated in coordination of the study and reviewed the medical records. Y Iinuma, Y. Ito, and SI participated in the design of the study, coordination, and manuscript preparation. MS, AM, MN, and ST participated in the genetic studies. All authors read and approved the final manuscript.

## Pre-publication history

The pre-publication history for this paper can be accessed here:

http://www.biomedcentral.com/1471-2334/11/76/prepub
